# Circadian Rhythm in Kidney Tissue Oxygenation in the Rat

**DOI:** 10.3389/fphys.2017.00205

**Published:** 2017-04-06

**Authors:** Tonja W. Emans, Ben J. Janssen, Jaap A. Joles, C. T. Paul Krediet

**Affiliations:** ^1^Department of Internal Medicine, Academic Medical Center at the University of AmsterdamAmsterdam, Netherlands; ^2^Department of Nephrology and Hypertension, University Medical Center UtrechtUtrecht, Netherlands; ^3^Department of Pharmacology and Toxicology, Maastricht UniversityMaastricht, Netherlands

**Keywords:** kidney oxygenation, circadian rhythm, renal cortex, renal medulla, hypertension, hypoxia

## Abstract

Blood pressure, renal hemodynamics, electrolyte, and water excretion all display diurnal oscillation. Disturbance of these patterns is associated with hypertension and chronic kidney disease. Kidney oxygenation is dependent on oxygen delivery and consumption that in turn are determined by renal hemodynamics and metabolism. We hypothesized that kidney oxygenation also demonstrates 24-h periodicity. Telemetric oxygen-sensitive carbon paste electrodes were implanted in Sprague-Dawley rats (250–300 g), either in renal medulla (*n* = 9) or cortex (*n* = 7). Arterial pressure (MAP) and heart rate (HR) were monitored by telemetry in a separate group (*n* = 8). Data from 5 consecutive days were analyzed for rhythmicity by cosinor analysis. Diurnal electrolyte excretion was assessed by metabolic cages. During lights-off, oxygen levels increased to 105.3 ± 2.1% in cortex and 105.2 ± 3.8% in medulla. MAP was 97.3 ± 1.5 mmHg and HR was 394.0 ± 7.9 bpm during lights-off phase and 93.5 ± 1.3 mmHg and 327.8 ± 8.9 bpm during lights-on. During lights-on, oxygen levels decreased to 94.6 ± 1.4% in cortex and 94.2 ± 8.5% in medulla. There was significant 24-h periodicity in cortex and medulla oxygenation. Potassium excretion (1,737 ± 779 vs. 895 ± 132 μmol/12 h, *P* = 0.005) and the distal Na^+^/K^+^ exchange (0.72 ± 0.02 vs. 0.59 ± 0.02 *P* < 0.001) were highest in the lights-off phase, this phase difference was not found for sodium excretion (*P* = 0.4). It seems that oxygen levels in the kidneys follow the pattern of oxygen delivery, which is known to be determined by renal blood flow and peaks in the active phase (lights-off).

## Introduction

Endogenous timing mechanisms have evolved to adapt to environmental changes imposed by alternating periods of light and darkness. Twenty-four hours patterns in sleep, activity, food intake, and excretion are well-known representatives of such physiological homeostatic adaptations. Via the kidneys, these homeostatic mechanisms maintain constancy of the bodies' extracellular fluid compartment throughout the 24-h cycle.

In mammals, diurnal variations in urinary volume and electrolyte excretion are the best studied features of 24-h renal rhythm. Urinary excretion of water, sodium, and potassium peak during the active period of the day when intake is also highest (Cohn et al., 1970; Hilfenhaus, 1976; Pons et al., 1996; Zhang et al., 2015). Concomitantly, the activity of the renin-angiotensin-aldosterone system (RAAS), which is a major regulator of blood pressure, appears in a circadian fashion in rodents (Hilfenhaus, 1976), and humans (Armbruster et al., 1975; Mahler et al., 2015). Herein, plasma aldosterone levels peak just before the activity phase and are inverted by reversal of the light-dark cycle (Hilfenhaus, 1976).

Disturbance of the circadian blood pressure pattern, exposed as a non-dipping profile at night, is associated with hypertension and nephropathy (Fukuda et al., 2006; Sachdeva and Weder, 2006). Sleep problems have been reported in almost 80% of end-stage renal disease patients (Koch et al., 2009), and a disturbed blood pressure pattern is associated with higher risk for chronic kidney disease (CKD) (Portaluppi et al., 1991). Not surprisingly, restoration of the dipping profile during the inactive phase has been a target of interest for anti-hypertensive chronotherapy (Simko and Pechanova, 2009). Timed-inhibition of the renin angiotensin system can be used to suppress the rise in blood pressure upon awakening (Oosting et al., 1999). Disturbed renal sodium transport seems to be linked to abnormal circadian blood pressure profiles (Fujii et al., 1999).

Troughs in the excretion patterns of electrolytes typically occur during the resting or sleeping phase of the 24-h cycle. Assuming that the kidneys consume most energy and oxygen on sodium transport (Brezis et al., 1994), one could hypothesize that tissue oxygen concentration (pO_2_) in renal tissue is lowest when sodium reabsorption activity is highest. On the other hand, the increased oxygen use in the kidney may be fully matched by increased oxygen delivery because 24-h variations in blood pressure and renal blood flow coincide with periods of highest excretion (Pons et al., 1996). Normal kidney oxygenation is crucial as disturbed pO_2_ within the kidneys has been linked to the progression of CKD (Evans et al., 2013). Data on 24-h variations in oxygenation are lacking because, until recently, it was not possible to measure kidney oxygenation continuously. To answer the question whether 24-h variations in renal function are associated with synchronous variations in renal oxygenation, pO_2_ levels in the kidney were continuously monitored in healthy rats by a telemetry based technique for 5 consecutive days. Additionally, 24-h variations in tissue oxygenation were compared for renal cortical and medullary tissue and linked to the magnitude of day/night differences in water and food intake, and urinary water and electrolyte excretion. Very recently, Adamovich et al. described that oxygen levels may adjust the timing of the internal circadian clock (Adamovich et al., 2017). They showed that in the rat kidney (cortex) a circadian rhythm in pO_2_ levels can be detected. In this study we further substantiate these finding and expand this to both the medullary and cortical region in the kidney.

## Methods

### Animals

Experiments were conducted in male Sprague Dawley rats (250–300 grams, supplier: Charles River). All procedures were approved by the Animal Ethics Committee of University of Utrecht (DEC 2014.II.03.015) and were in accordance with the Dutch Codes of Practice for the Care and Use of Animals for Scientific Purposes. All animals were kept on a 12 h light/dark cycle with lights-on at 6 a.m. (ZT 0), and lights-off at 6 p.m. (ZT 12). Rats had access to water and standard rat chow (contains 0.3% Na^+^ and 0.69% K^+^) *ad libitum*. To promote animal welfare and normal physiological activity around the clock, the rats were cohoused. Only during urine collection for electrolyte analysis were the rats housed individually for 24 h.

### System overview

The telemetry based technique to measure oxygenation in the kidney has been described in detail (Emans et al., 2016; Koeners et al., 2016). In summary, oxygen sensitive carbon paste electrodes were implanted in the right kidney, either in the cortex (*n* = 7) or medulla (*n* = 9). The kidney was exposed via laparotomy. The telemeter (TR57Y, Millar, Houston, US) was placed in the rat abdomen. After 2 weeks of complete recovery and stabilization of oxygen signal, 24-h oscillations in pO_2_ were recorded continuously. In a third group, blood pressure telemeters (TRM54P, Millar, Houston, US) were implanted in the abdominal aorta (*n* = 8).

### Analysis

After subtraction of the off-set value, original pO_2_ data were filtered with a 25 Hz low-pass digital filter. Artifacts were removed when the 1st order derivative exceeded a threshold of 5 nA/s, as described previously (Emans et al., 2016). To describe the 24-h rhythm, 1 h average pO_2_-values were calculated. These hourly averages were used to determine the mean pO_2_ level over the 5 consecutive days and were then re-expressed relatively to this 5-day mean value (MESOR). The Cosinor method was applied to determine the amplitude and phase of the oscillation in the pO_2_ signal (Refinetti et al., 2007). Twenty-four hours rhythmicity was determined when the amplitude of the fitted curve was significantly >0. For blood pressure and heart rate, absolute values were used.

### Electrolyte excretion

Rats were individually housed in metabolic cages for 24 h (*n* = 13) to determine water and food intake and to collect urine. Urine was sampled in epochs of 12 h starting at 6 p.m. (lights-off/active phase) and continued at 6 a.m. (lights-on/resting phase). In these 12-h urine samples sodium and potassium concentrations were determined by flame photometry (Model 420, Sherwood, UK). Urinary creatinine was determined by DiaSys Kit (DiaSys Diagnostic Systems, Holzheim, Germany). Distal sodium/potassium exchange was quantified as kaliuresis/(natriuresis + kaliuresis; Hene et al., 1984).

### Statistics

Data are expressed as mean ± SEM. The data collected during active respectively resting phase were compared by paired Student's *t*-test. Differences were considered significant when *p* < 0.05.

## Results

**Figure 1:**
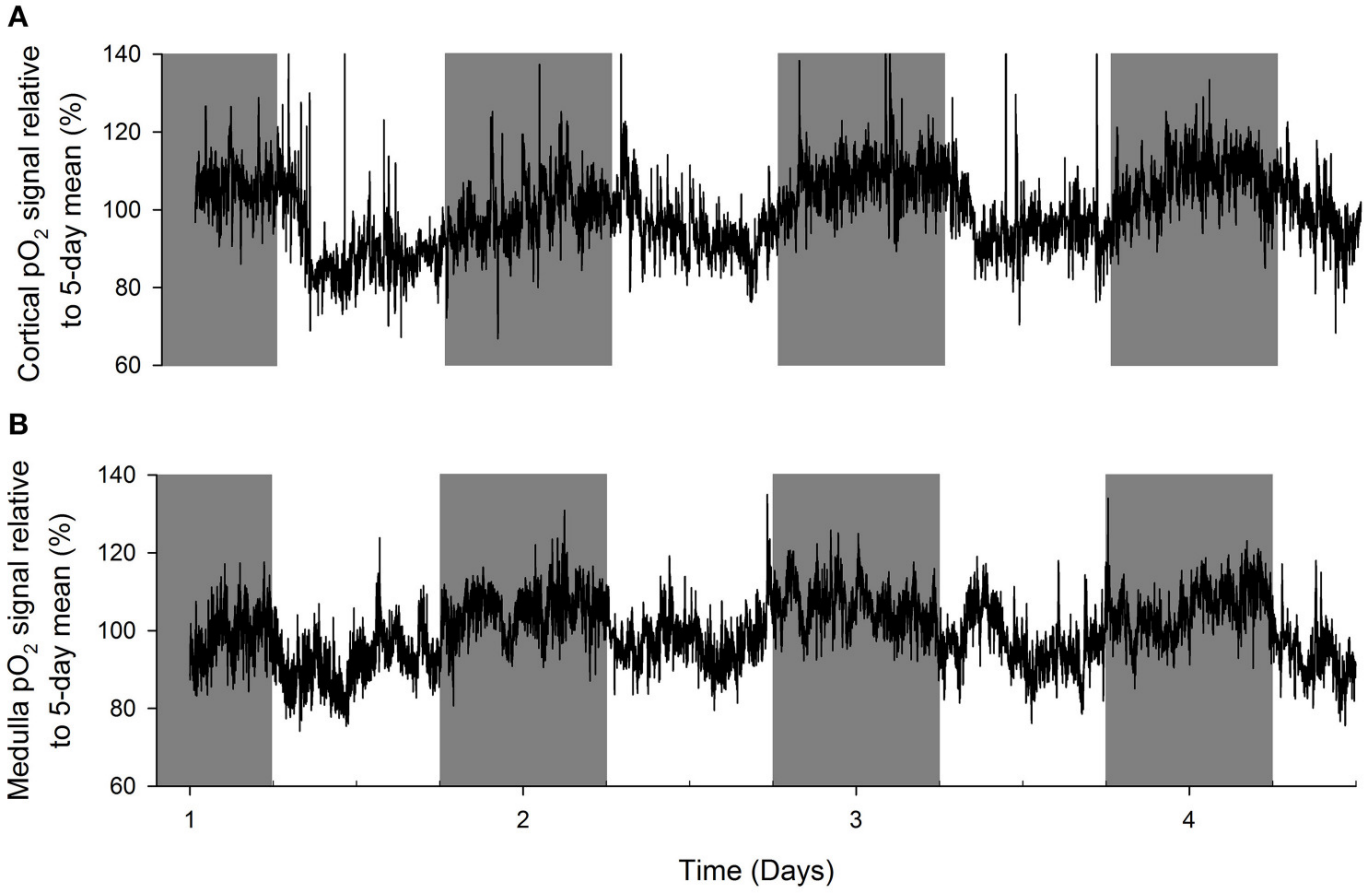
**Representative examples of original recordings (A)** in a rat with an oxygen sensor placed in the cortex of the kidney and **(B)** in another rat with the probe in the renal medulla. Data are 10 second averages during consecutive days. Note that in both cortex and medulla, tissue oxygenation peaks during the lights-off (active) period of the day (marked in gray). Troughs in tissue pO_2_ are visible in the lights-on (resting) period.

**Figure 2:**
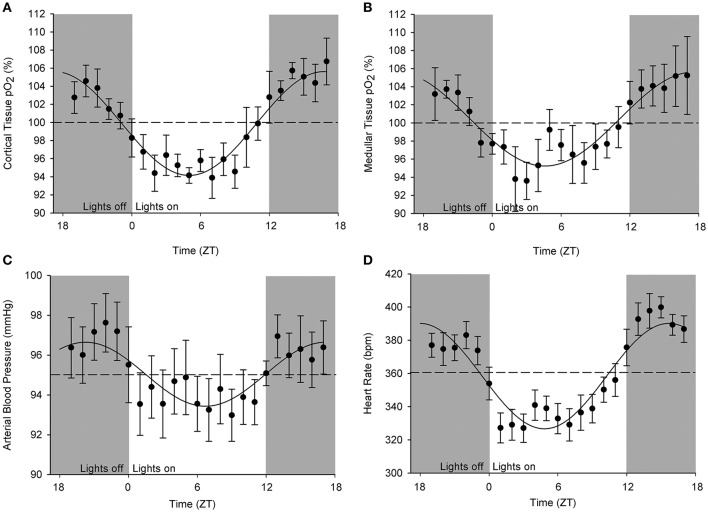
**Cosinor analysis of the averaged circadian rhythms in kidney oxygenation, blood pressure, and heart rate**. Data are plotted as hourly mean values ± SEM as recorded over 5 days in each rat relatively to the overall mean value (= MESOR, indicated by the dotted line). Note that different rats were used for obtaining oxygenation in **(A)** cortical and **(B)** medullary pO_2_ as well as for the **(C)** blood pressure. **(D)** Heart rate values were derived from blood pressure measurements. A significant circadian rhythm was assessed when the amplitude of the fitted curve was statistically >0, see Table 1.

An original tracing of 3.5-day consecutive recording of cortical pO_2_ levels is depicted in Figure 1A. An example of a tracing obtained with the probe in the medulla is presented in Figure 1B. On visual inspection of the raw telemetric data, oxygen levels in both the cortex and medulla peaked during the lights-off period in these nocturnally active rats, while trough values were usually found during the lights-on or resting period of the day. The 5-day mean was set at 100% pO_2_. Quantification of these observations by the Cosinor analyses revealed that during the lights-off phase, oxygen levels increased to 105.3 ± 2.1 and 105.2 ± 3.8% in renal cortex and medulla, respectively. During the lights-on phase, oxygen levels decreased to 94.6 ± 1.4% in cortex and 94.2 ± 8.5% in medulla, relatively to the 5-day mean (Table 1). The mean amplitude of the fitted curve for pO_2_ rhythmicity tended to be larger in cortex than in medulla (5.8 vs. 4.9%), although this difference was not significant (Figure 2). Ninety-five percentage Confidence intervals (95% CI) of both cortex and medulla oxygenation did not exceed the MESOR. Twenty-four hours blood pressure and heart rate rhythms were in phase with those occurring in pO_2_.

**Table 1:** **Circadian parameters in oxygenation, blood pressure, and heart rate**.

	**MESOR**	**Amplitude**	**Lights-off peak (active)**	**Lights-on peak (rest)**	**Acrophase (ZT h)**	**Robustness (%)**
Oxygenation cortex (%)	100.0 (99.3–100.7)	5.8 (4.7–6.8)^*^	105.3 ± 2.1	94.6 ± 1.4	16.9 (16.3–17.4)	93.3
Oxygenation medulla (%)	100.0 (98.8–101.2)	4.9 (3.6–6.3)^*^	105.2 ± 3.8	94.2 ± 8.5	16.9 (16.0–17.7)	86.2
Blood pressure (mmHg)	95.5 (94.6–95.8)	1.6 (0.8–2.4)^*^	97.3 ± 1.5	93.5 ± 1.3	18.9 (17.6–20.2)	69.9
Heart rate (bpm)	361 (356–365)	33 (28–38)^*^	394 ± 8	328 ± 9	16.9 (16.2–17.6)	87.4

Data were analyzed by cosinor analysis (period = 24 h), lighting schedule; lights-on at 6 a.m. (ZT0), and lights-off at 6 p.m. (ZT12). Circadian rhythm-adjusted mean, MESOR. Peak time of cosine function, Acrophase. Percentage of variance accounted for by the computed rhythm, Robustness. (95% CI except for peak values mean ± SEM),

* *P < 0.01 vs. zero amplitude = no rhythm*.

**Figure 3:**
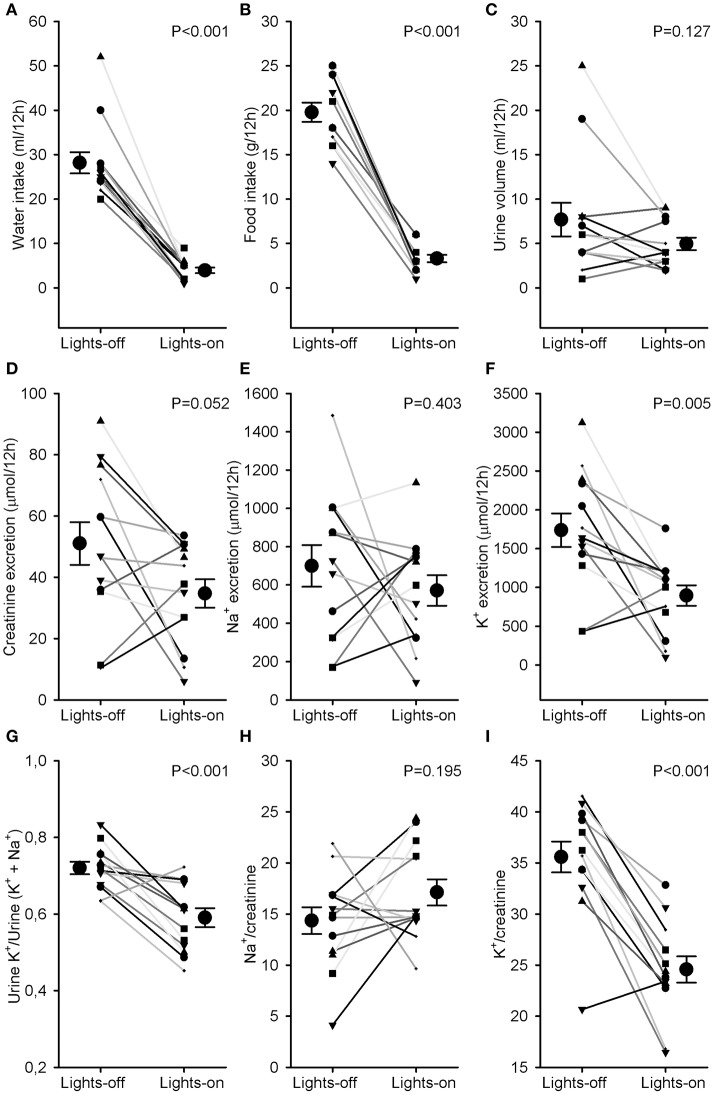
**Water and food intake and urine analysis**. Rats were individually housed in metabolic cages for 24 h. Urine was collected in 2 samples, one during the lights-off (active) and one during the lights-on (resting) period. Individual data and mean ± SEM are indicated for **(A)** water intake, **(B)** food intake, **(C)** urine volume, **(D)** creatinine excretion, **(E)** Na^+^ excretion, **(F)** K^+^ excretion, **(G)** Urine K^+^/Urine (K^+^ + Na^+^) as an estimate of distal Na^+^/K^+^ exchange, **(H)** Na^+^/creatinine, and **(I)** K^+^/creatinine. Lights-on/off differences were compared with paired Student's *t*-tests.

Water and food intake were significantly higher during the lights-off phase than during the lights-on phase (28 ± 2 vs. 4 ± 1 ml and 20 ± 1 vs. 3 ± 1 g, *P* < 0.001, Figures 3A,B). Urine volume did not differ much between lights-off and lights on (Figure 3C). Creatinine excretion tended to increase during the lights-off vs. lights-on phase (*P* = 0.052, Figure 3D). There was no phase difference for urinary sodium excretion or Na^+^/creatinine (Figures 3E,H), but urinary potassium excretion and K^+^/creatinine were increased during the 12 h lights-off vs. the lights on period (*P* < 0.01, Figures 3F,I). Distal Na^+^/K^+^ exchange (kaliuresis/natriuresis + kaliuresis) was increased in the lights-off period (*P* < 0.001, Figure 3G).

## Discussion

Normotensive rats display a significant diurnal rhythmicity in renal oxygenation (Adamovich et al., 2017). In this study we dissected the rhythmicity of cortical and medullary oxygenation, that both follow a diurnal pattern. Using telemetric techniques, peak values in tissue oxygenation were found during the lights-off period when renal excretion of electrolytes was highest. Trough values in renal pO_2_-values were observed during the lights-on period when excretion patterns are minimal. These data suggest that the circadian rhythm in (both cortical and medullar) pO_2_ is mainly the result of a 24 variation in oxygen delivery to the kidneys.

In rats, cardiac output and blood pressure are highest during the lights-off period (Oosting et al., 1997), when rats display highest locomotor activity and eat and drink the most. Assuming that during normal activity renal blood flow is stable at approximately 20% of cardiac output, oxygen delivery is highest to this organ during the active phase. While direct renal blood flow measurements are not available over 24 h, previous studies in rats using inulin and p-aminohippuric acid clearance have repeatedly (Pons et al., 1996) shown that GFR and RBF indeed peak during the lights-off period. This corroborates the hypothesis that oxygen delivery is the most important determinant of this circadian pattern in oxygenation. Recently, 24-h oxygen recordings in the kidneys have been obtained in sheep (Calzavacca et al., 2015). In that study, a clear 24-h pattern in tissue oxygenation of the kidneys was absent. RBF and tissue perfusion did not show a circadian fluctuation either. Presumably, there was a suppression of normal locomotor activity in these sheep because they were housed in metabolic cages. In the current study, we co-housed the rats to facilitate normal social behavior and thereby normal locomotor activity. An alternative explanation for the discrepancy between our observations and those in sheep may be that ruminants have a less pronounced fasting phase than omnivorous species such as rats and that that the delivery of nutrients is more constant than in diurnal active species such as rats. While studying mechanisms of 24-h variation in potassium excretion, Steel et al. found that the bulk of potassium excretion was determined by food intake (delivery) rather than the flow (Steele et al., 1994). This suggests that peak oxygen levels in the kidneys may also occur in parallel with delivery patterns of certain nutrients, waste products, or electrolytes. Future studies are needed to sort out cause and consequence of such associations. Very recently, a similar daily pattern in pO_2_ in the kidney was briefly described in rodents. Peak values were found during lights-off, when oxygen consumption rate was highest as well (Adamovich et al., 2017).

In rats ANGII and Aldosterone peak during the lights-on period, when electrolyte excretion is lowest (Hilfenhaus, 1976; Lemmer et al., 2000; Naito et al., 2009). These hormones stimulate tubular sodium re-absorption during the lights-on period and thereby determine oxygen use. Presumably, this is an evolutionary mechanism to retain fluids when water intake is low. RBF and GFR also exhibit 24 h periodicity, peaks during lights-off and troughs during lights-on (Pons et al., 1996). Important genes related to renal sodium and water transport, like NHE, aquaporin 2 and 4, have been linked to circadian expression (Saifur Rohman et al., 2005). A lower kaliuresis and distal Na^+^/K^+^ exchange at the time of a decline in tissue oxygenation at rest suggest that oxygen consumption *per se* is not contributing to the pattern of renal oxygen content in our study.

The variations in arterial blood pressure and heart rate throughout the day have been studied in detail in healthy, chronically instrumented rats (Henry et al., 1990; Janssen et al., 1992; Teerlink and Clozel, 1993; van den Buuse, 1994). The variation between the nightly peaks and daily troughs are less pronounced in our study than reported by some others. This is probably caused by the fact that the light dark cycle in the animal room corresponded with real day and night making it possible that researcher or care taker-induced minor disturbances may have occurred in the recordings during the daily resting phase of the animals thereby underestimating the current 24-h amplitude in blood pressure oscillation. Reversing the experimental light/dark cycle would probably not have diminished the current 24-h amplitudes but actually magnified them. This may also apply for pO_2_-values. We decided to set the 5-day mean at 100% pO_2_ for each animal to allow inter-animal comparison (Emans et al., 2016). The between animal comparison would decrease the sensitivity by introducing a large SD between animals and obscuring day/night variation within one animal. Another technical limitation (inherent to studying small rodents) was that we were not able to record RBF variations. However, our setting does not interfere with natural behavior and physiological processes of the nocturnal animals. The rats were unrestrained and cohoused, which allows them full activity at night, accompanied by higher heart rates and probably a higher RBF as well.

Oxygen and arterial pressure assessment could not be performed in the same animal. Cortex and medulla recordings were also performed in separate animals. However, the rhythms were consistent within each of the three groups, suggesting that extrapolation of the results to the full set is acceptable. The animals were fully acclimatized toward the 12:12 light dark cycle in our facility. Our assessment of natriuresis may have been affected by our chosen 12-h sampling period, because sodium excretion can peak just before the light phase (Roelfsema et al., 1980; Pons et al., 1996). Due to ethical and technical issues, we decided not to acclimatize our rats to metabolic cage housing. This may have interfered with the excretion values calculated from collected urine. Other researchers, who applied an equilibration period in metabolic cages for 2–3 days (Nikolaeva et al., 2012; Johnston et al., 2016) did find significant differences in urine sodium excretion in rodents. However, since diurnal effects were prominent for fluid and food intake, urine flow, kaliuresis, and distal Na/K exchange, this suggests that, if anything, these diurnal differences would have been even more marked in acclimatized rats.

The kidney has more clock regulated genes than most other organs (Gumz, 2016). It has also been suggested that every cell type in the kidney follows its own circadian clock (Tokonami et al., 2014). Probably cortex and medulla follow their own circadian pattern as well. However, we did not find a different pattern in oxygenation between cortex and medulla. Our data suggest that the kidney may be more vulnerable to hypoxia during sleep. Actions that make the kidneys hypoxic in general could lead to damage during the resting phase, when the oxygen concentrations are already somewhat lower. This could be relevant in the pathogenesis of diseases that are associated with low oxygenation at night, such as obstructive sleep apnea and the associated progression of renal disease. One could argue that diseases associating with kidney hypoxia, for instance CKD (Evans et al., 2013) and diabetes (Franzen et al., 2016), could be more progressive during the resting phase. On the other hand, our data may also provide a new look at the association of neglecting to align with the inner circadian clock (e.g., in shift workers) with the development of hypertension and CKD (Lieu et al., 2012). Furthermore, low oxygen levels at rest could contribute to the non-dipping profile and hypertension, because low levels of oxygen in the kidneys may not allow a normal decline in MAP and RBF during rest.

In conclusion, the circadian rhythm of regional kidney oxygenation that we describe, is a new phenomenon that provides further research opportunities for the onset and progression of hypertension and CKD.

## Author contributions

TE, BJ, JJ, CK concept and design of research; TE performed experiments; TE, BJ, JJ, CK analyzed data; TE, BJ, JJ, CK interpreted results of experiments; TE prepared figures; TE, BJ drafted manuscript; TE, BJ, JJ, CK edited and revised manuscript; TE, BJ, JJ, CK approved final version of manuscript; TE, BJ, JJ, CK ensure integrity.

## Funding

CK is supported by the Netherlands Organization for Health Research (ZonMw, Clinical Fellowship 40007039712461) and by the Dutch Kidney Foundation (Project KJPB 12.029). BJ is supported by the Dutch Kidney Foundation (Grant 16OI21). This support is gratefully acknowledged.

### Conflict of interest statement

The authors declare that the research was conducted in the absence of any commercial or financial relationships that could be construed as a potential conflict of interest.

## Abbreviations

CKD: chronic kidney disease
GFR: glomerular filtration rate
HR: heart rate
MAP: mean arterial pressure
MESOR: circadian rhythm adjusted mean
pO_2_: tissue oxygen concentration
RAAS: renin-angiotensin-aldosterone system
RBF: renal blood flow.
